# SOCIAL STRUCTURAL DISPARITIES IN DENTAL CARE UTILIZATION AMONG ADULTS IN CHINA’S MEGACITIES

**DOI:** 10.1093/geroni/igad104.0052

**Published:** 2023-12-21

## Abstract

This study utilized data from the “2019 New Era and Living Conditions in Megacities Survey” to investigate social structural disparities in dental care utilization among adults aged 18-65 from 10 megacities in China. Logistic regressions were conducted to examine the relationships between social structural factors, such as hukou status, occupational status, migrant status, and city of residence, and dental care utilization. Margins post estimation was used to explore whether the social structural disparities in dental care utilization were associated with changes in oral health status. Results showed that only 22.36% of participants had at least one dental visit per year. Having urban hukou as compared with rural hukou (OR, 1.76; 95% CI, 1.47-2.12), working in state-owned enterprise as compared with in other enterprises (OR, 1.34; 95% CI, 1.14-1.57), and living in more developed cities as compared with in less developed cities (OR, 1.48; 95% CI, 1.27-1.72) were associated with a higher likelihood of regular dental visits. Additionally, participants with urban hukou and those living in more developed cities were more likely to have regular dental visits when their oral health was good, such as when they had full natural teeth. This study highlights social structural disparities in oral health behaviors among adults in China’s megacities. The findings suggest that efforts should be made to improve dental care utilization by promoting public messaging about oral health knowledge, expanding dental care coverage, and improving access to dental care services in China.

